# Supplementary material for: Increased reactivity of the paraventricular nucleus of the hypothalamus and decreased threat responding in male rats following psilocin administration
Source: Nat Commun. 2024 Jun 22;15:5321. doi: 10.1038/s41467-024-49741-9 (PMC11193716; doi:10.1038/s41467-024-49741-9)
Supplement: Supplementary file 1 — Supplementary Information [file 41467_2024_49741_MOESM1_ESM.pdf]

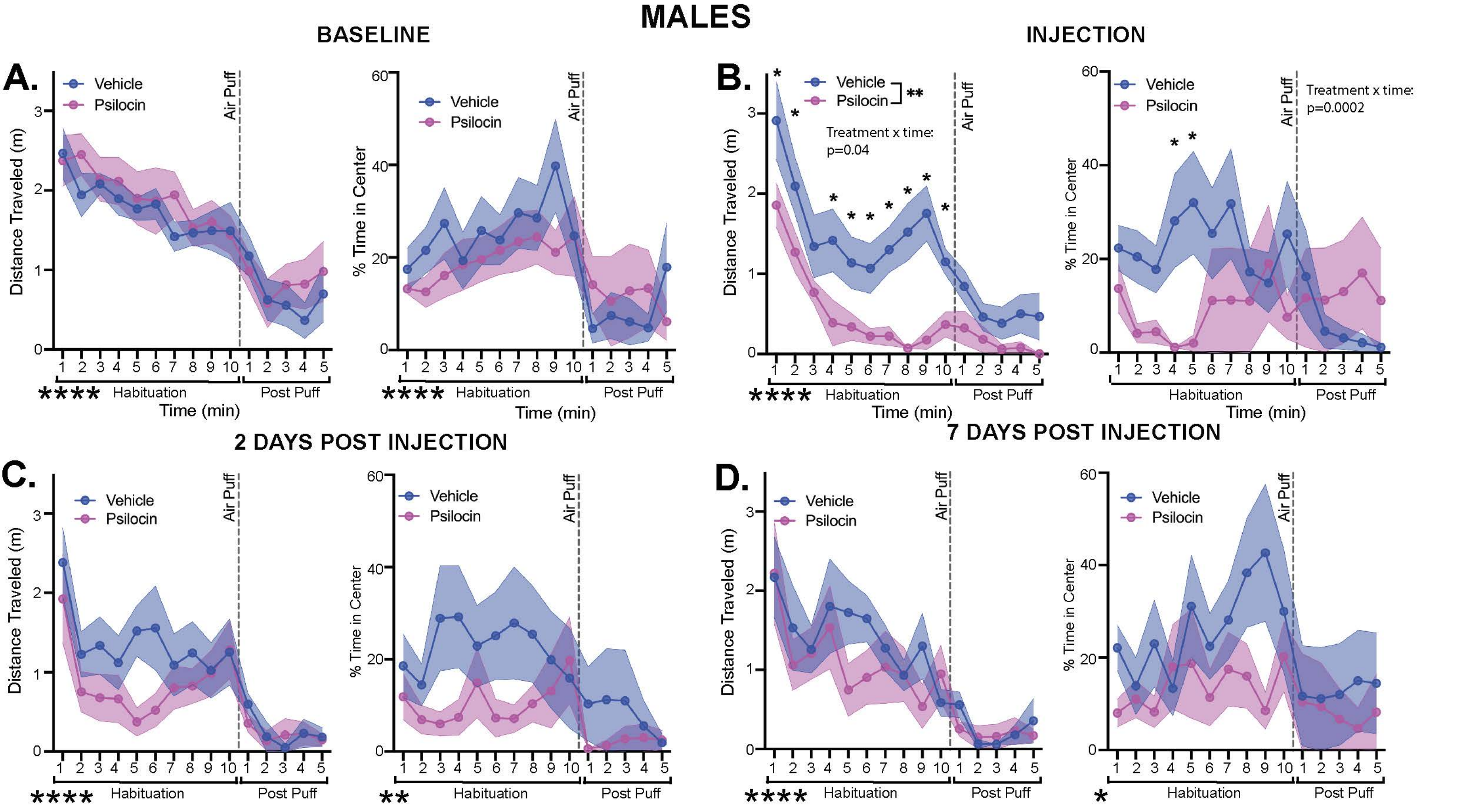

Supplementary Figure 1 (S1). Acute and prolonged effects of psilocin on general behavior in males. Psilocin n=9, Vehicle n=9; (A) Male baseline locomotor (left) and time spent in center (right) figures showing group averages across a time course across habituation and post-air-puff inter-stimulus-interval (ISI). A 2-way ANOVA revealed a main effect of time (p<0.0001) in both locomotor and time spent in center. Each data point represents a subject. Shaded area surrounding mean line represents +/- S.E.M. (B) Male day of injection locomotor (left) and time spent in center (right) figures showing group averages across a time course across habituation and post-air-puff inter-stimulus-interval (ISI). A 2-way ANOVA revealed a significant treatment x time interaction (p=0.0405), main effect of time (p<0.0001) and treatment (p=0.0021) in locomotion and a significant treatment x time interaction (p=0.0002) in time spent in center. Šidák multiple comparisons found significant differences in locomotion at minutes 1 (p=0.0033), 2 (p=0215), 4 (p=0.0042), 5 (p=0.0255), 6 (p=0.0180), 7 (p=0.0027), 8 (p<0.0001), 9 (p<0.0001), and 10 (p=0.0180) of the habituation period. In time spent in center there were significant differences at minutes 4 (p=0.0219) and 5 (p=0.0110). Each data point represents a subject. Shaded area surrounding mean line represents +/- S.E.M. (C) Male 2-days post-injection locomotor (left) and time spent in center (right) figures showing group averages across a time course across habituation and post-air-puff inter-stimulus-interval (ISI). A 2-way ANOVA revealed a main effect of time (locomotion: p<0.0001, time in center: p=0.0054). Each data point represents a subject. Shaded area surrounding mean line represents +/- S.E.M. Uncorrected Fisher's LSD identified the difference between groups at 2 minutes during the post puff ISI as driving this effect; corrected post hoc comparisons did not reach statistical significance. (D) Male 7-days post-injection locomotor (left) and time spent in center (right) figures showing group averages across a time course across habituation and post-air-puff inter-stimulus-interval (ISI). A 2-way ANOVA revealed a main effect of time (locomotion: p<0.0001, time in center: p=0.0170) Each data point represents a subject. Shaded area surrounding mean line represents +/- S.E.M. *p<0.05, **p<0.01, *** p<0.001, **** p<0.0001.

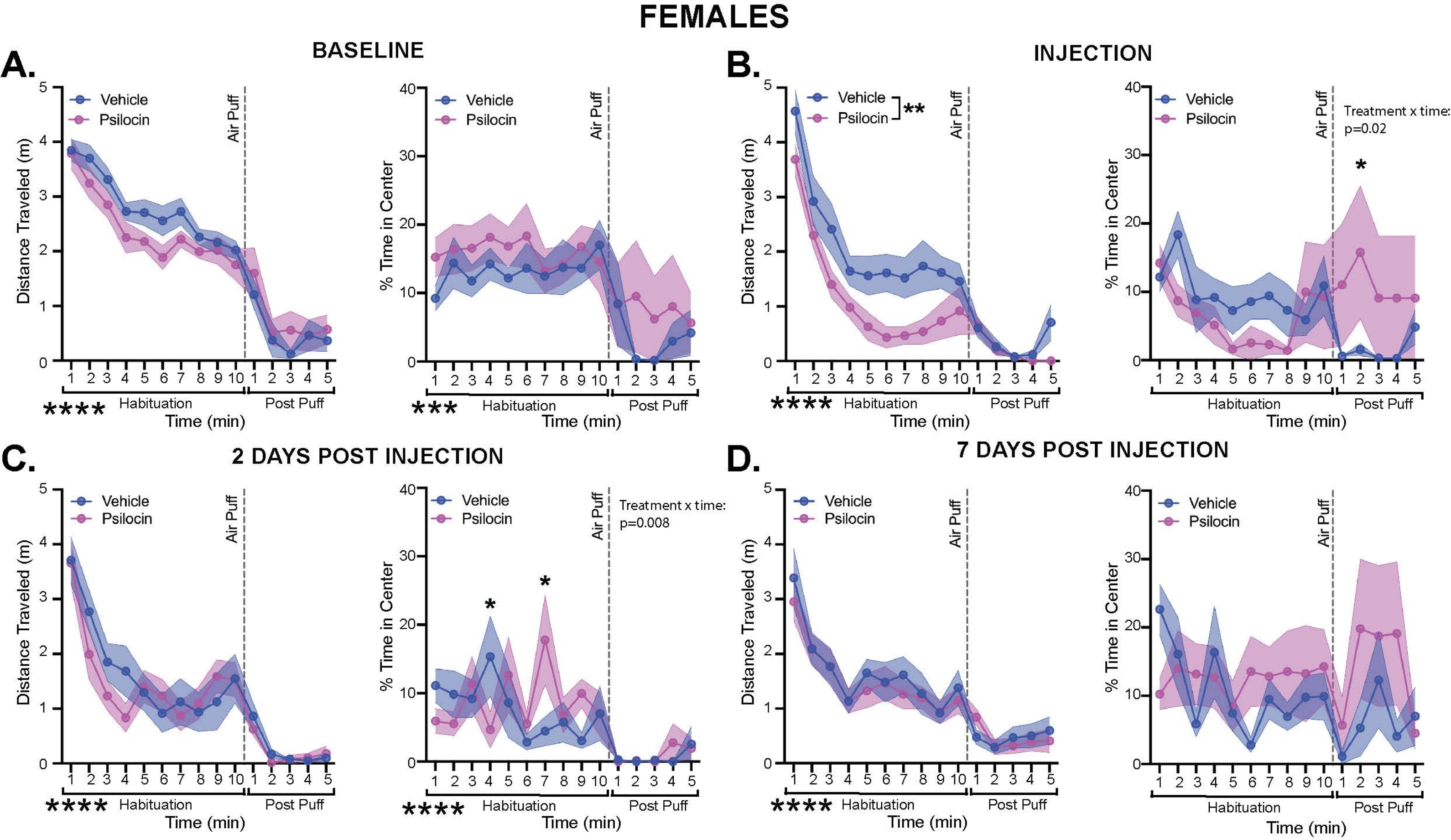

Supplementary Figure 2 (S2). Acute and prolonged effects of psilocin on general behavior in females. Psilocin n=11, Vehicle n=10; (A) Baseline locomotor (left) and time spent in center (right) figures showing group averages across a time course across habituation and post-air-puff inter-stimulus-interval (ISI). A 2-way ANOVA revealed a main effect of time (locomotion: $p<0.0001$, time in center: $p=0.0002$). Each data point represents a subject. Shaded area surrounding mean line represents +/- S.E.M. (B) Day of injection locomotor (left) and time spent in center (right) figures showing group averages across a time course across habituation and post-air-puff inter-stimulus-interval (ISI). A 2-way ANOVA revealed a main effect of time ($p<0.0001$) and treatment ($p=0.0075$) in locomotion and a treatment x time interaction ($p=0.0244$) in time spent in center. Šidák multiple comparisons revealed significant differences at minute 2 following air-puff ($p=0.0433$). Each data point represents a subject. Shaded area surrounding mean line represents +/- S.E.M. (C) 2-days post-injection locomotor (left) and time spent in center (right) figures showing group averages across a time course across habituation and post-air-puff inter-stimulus-interval (ISI). A 2-way ANOVA revealed a main effect of time ($p<0.0001$) for locomotion and a treatment x time interaction ($p=0.0079$) and main effect of time ($p<0.0001$) for time spent in center. Šidák multiple comparisons revealed significant differences at minute 4 ($p=0.0115$) and 7 ($p=0.0017$) during habituation. Each data point represents a subject. Shaded area surrounding mean line represents +/- S.E.M. (D) Female 7-days post-injection locomotor (left) and time spent in center (right) figures showing group averages across a time course across habituation and post-air-puff inter-stimulus-interval (ISI). A 2-way ANOVA revealed a main effect of time ($p<0.0001$) for locomotion. Each data point represents a subject. Shaded area surrounding mean line represents +/- S.E.M. *$p<0.05$, **$p<0.01$, *** $p<0.001$, **** $p<0.0001$.

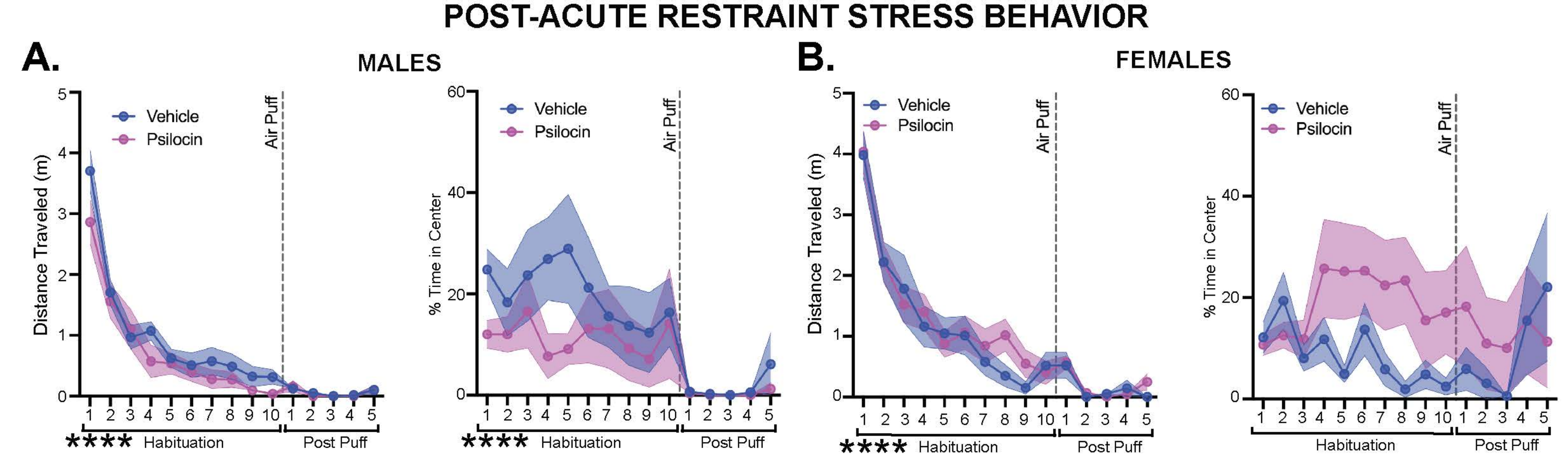

Supplementary Figure 3 (S3). Effects of psilocin on general behavior following acute restraint stress. Males: Psilocin n=6, Vehicle n=7; Female: Psilocin n=9, Vehicle n=9; (A) Male post-restraint locomotor (left) and time spent in center (right) figures showing group averages across a time course across habituation and post-air-puff inter-stimulus-interval (ISI). A 2-way ANOVA revealed a main effect of time ($p<0.0001$) for locomotion and time in center. Each data point represents a subject. Shaded area surrounding mean line represents +/- S.E.M. (B) Female post-restraint locomotor (left) and time spent in center (right) figures showing group averages across a time course across habituation and post-air-puff inter-stimulus-interval (ISI). A 2-way ANOVA revealed a main effect of time ($p<0.0001$) in locomotion. Each data point represents a subject. Shaded area surrounding mean line represents +/- S.E.M. *$p<0.05$, **$p<0.01$, *** $p<0.001$, **** $p<0.0001$.

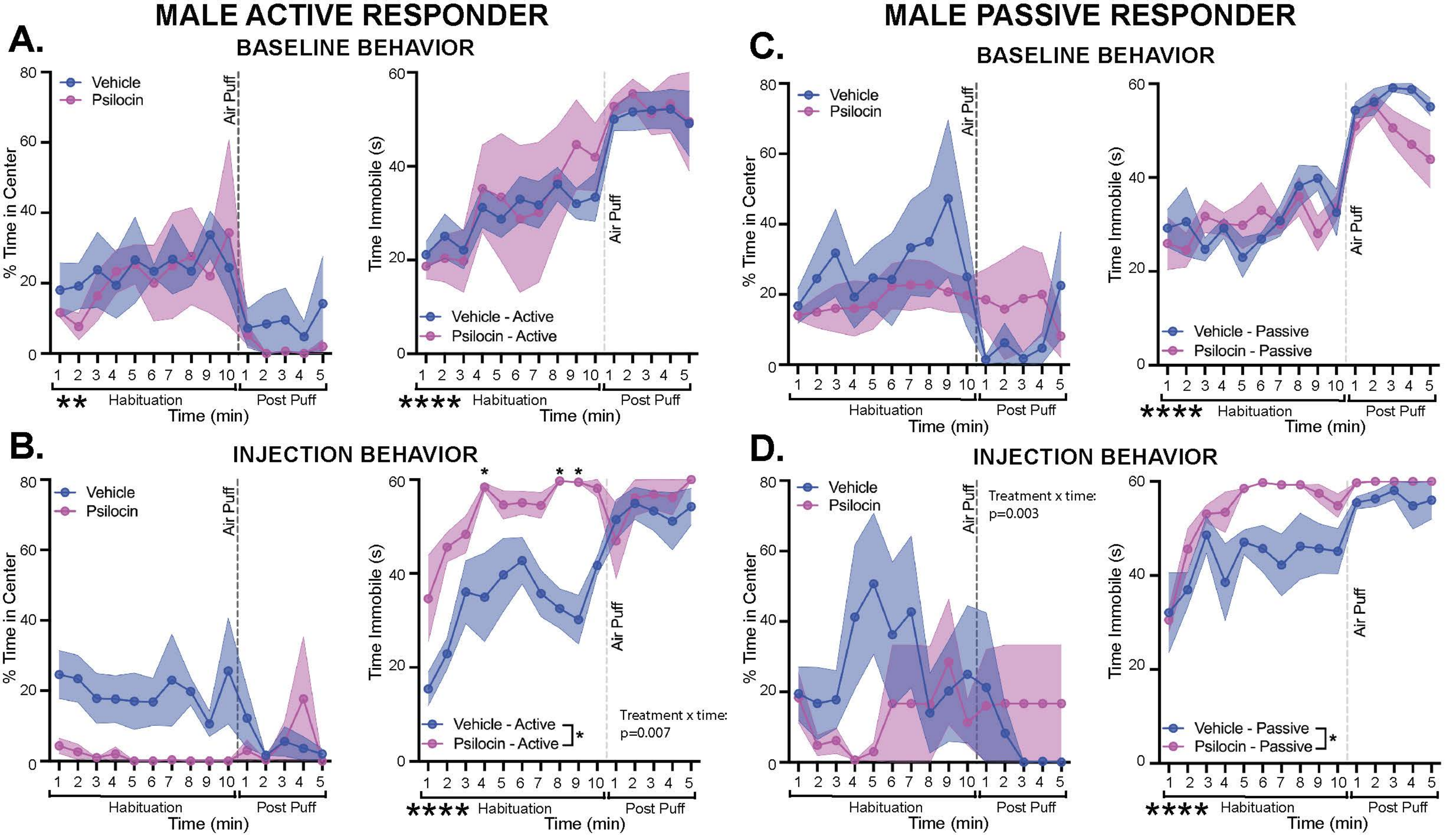

Supplementary Figure 4 (S4). Effects of psilocin on general behavior between active and passive responding males. Active: Psilocin group n=3, Vehicle group n=5; Passive: Psilocin group n=6, Vehicle group n=4; (A) Male active responders: baseline time spent in center (left) and time immobile (right) figures showing group averages across a time course across habituation and post-air-puff inter-stimulus-interval (ISI). A 2-way ANOVA revealed a main effect of time (time in center: $p=0.0016$, immobility: $p<0.0001$). Each data point represents a subject. Shaded area surrounding mean line represents +/- S.E.M. (B) Male active responders: day of injection time spent in center (left) and time immobile (right) figures showing group averages across a time course across habituation and post-air-puff inter-stimulus-interval (ISI). A 2-way ANOVA revealed a treatment x time interaction in immobility ($p=0.0076$) and main effects of time ($p<0.0001$) and treatment ($p=0.0188$). Šidák multiple comparisons revealed significant differences at minutes 4 ($p=0.0404$), 8 ($p=0.0087$), and 9 ($p=0.0034$) during habituation. Each data point represents a subject. Shaded area surrounding mean line represents +/- S.E.M. (C) Male passive responders: baseline time spent in center (left) and time immobile (right) figures showing group averages across a time course across habituation and post-air-puff inter-stimulus-interval (ISI). A 2-way ANOVA revealed a main effect of time in immobility ($p<0.0001$). Each data point represents a subject. Shaded area surrounding mean line represents +/- S.E.M. (D) Male passive responders: day of injection time spent in center (left) and time immobile (right) figures showing group averages across a time course across habituation and post-air-puff inter-stimulus-interval (ISI). A 2-way ANOVA revealed a treatment x time interaction for time spent in center ($p=0.0034$) and a main effect of time ($p<0.0001$) and treatment ($p=0.0103$) for immobility. Each data point represents a subject. Shaded area surrounding mean line represents +/- S.E.M. *$p<0.05$, **$p<0.01$, *** $p<0.001$, **** $p<0.0001$.
