# Supplementary material for: Increased reactivity of the paraventricular nucleus of the hypothalamus and decreased threat responding in male rats following psilocin administration
Source: Nat Commun. 2024 Jun 22;15:5321. doi: 10.1038/s41467-024-49741-9 (PMC11193716; doi:10.1038/s41467-024-49741-9)
Supplement: Supplementary file 3 — Reporting Summary [file 41467_2024_49741_MOESM3_ESM.pdf]

nature portfolio

Corresponding author(s): Melissa Herman

Last updated by author(s): 5/26/2024

Reporting Summary

Nature Portfolio wishes to improve the reproducibility of the work that we publish. This form provides structure for consistency and transparency in reporting. For further information on Nature Portfolio policies, see our Editorial Policies and the Editorial Policy Checklist.

Statistics

For all statistical analyses, confirm that the following items are present in the figure legend, table legend, main text, or Methods section.

n/a	Confirmed	
☐	☒	The exact sample size (*n*) for each experimental group/condition, given as a discrete number and unit of measurement
☒	☐	A statement on whether measurements were taken from distinct samples or whether the same sample was measured repeatedly
☐	☒	The statistical test(s) used AND whether they are one- or two-sided<br>*Only common tests should be described solely by name; describe more complex techniques in the Methods section.*
☐	☒	A description of all covariates tested
☐	☒	A description of any assumptions or corrections, such as tests of normality and adjustment for multiple comparisons
☐	☒	A full description of the statistical parameters including central tendency (e.g. means) or other basic estimates (e.g. regression coefficient) AND variation (e.g. standard deviation) or associated estimates of uncertainty (e.g. confidence intervals)
☐	☒	For null hypothesis testing, the test statistic (e.g. *F*, *t*, *r*) with confidence intervals, effect sizes, degrees of freedom and *P* value noted<br>*Give P values as exact values whenever suitable.*
☒	☐	For Bayesian analysis, information on the choice of priors and Markov chain Monte Carlo settings
☒	☐	For hierarchical and complex designs, identification of the appropriate level for tests and full reporting of outcomes
☒	☐	Estimates of effect sizes (e.g. Cohen's *d*, Pearson's *r*), indicating how they were calculated

Our web collection on statistics for biologists contains articles on many of the points above.

Software and code

Policy information about availability of computer code

Data collection	Fiber photometry data were collected using the TDT RZ5 real time processing unit. Behavioral video data were collected through Anymaze.
Data analysis	Data analysis was conducted using Matlab 2023a and GraphPad Prism. Custom Matlab script was used.

For manuscripts utilizing custom algorithms or software that are central to the research but not yet described in published literature, software must be made available to editors and reviewers. We strongly encourage code deposition in a community repository (e.g. GitHub). See the Nature Portfolio guidelines for submitting code & software for further information.

Data

Policy information about availability of data

All manuscripts must include a data availability statement. This statement should provide the following information, where applicable:

- Accession codes, unique identifiers, or web links for publicly available datasets
- A description of any restrictions on data availability
- For clinical datasets or third party data, please ensure that the statement adheres to our policy

Data have been uploaded to Dryad and are available to reviewers during the process. Custom written Matlab scripts and subsequent data structures are available there and available to reviewers.
Effinger, Devin (Forthcoming 2024). Psychedelic drug effects on reactivity of the paraventricular nucleus of the hypothalamus and threat responding behavior [Dataset]. Dryad. https://doi.org/10.5061/dryad.3ffbg79qr

Research involving human participants, their data, or biological material

Policy information about studies with human participants or human data. See also policy information about sex, gender (identity/presentation), and sexual orientation and race, ethnicity and racism.

Reporting on sex and gender	N/A
Reporting on race, ethnicity, or other socially relevant groupings	N/A
Population characteristics	N/A
Recruitment	N/A
Ethics oversight	N/A

Note that full information on the approval of the study protocol must also be provided in the manuscript.

Field-specific reporting

Please select the one below that is the best fit for your research. If you are not sure, read the appropriate sections before making your selection.

☒ Life sciences ☐ Behavioural & social sciences ☐ Ecological, evolutionary & environmental sciences

For a reference copy of the document with all sections, see nature.com/documents/nr-reporting-summary-flat.pdf

Life sciences study design

All studies must disclose on these points even when the disclosure is negative.

Sample size	Sample size was determined based on previous work published in the lab and taking into consideration a prior power calculations.
Data exclusions	8 subjects were removed from the study due to lack of signal. 8 subjects were removed from behavioral analyses consisting of habituation due to camera malfunction.
Replication	PVN reactivity was confirmed and reproduced between a male and female cohort. Further replication of cohorts were not conducted.
Randomization	Groups were formed based on baseline responding to ensure that any changes in reactivity seen following drug exposure were not an artifact of differing baseline values.
Blinding	Experimenters were blinded for immunohistochemical analysis of tissue and manual behavioral scoring. Experimenter was not blinded to conditions during behavioral recording as measurements of PVN reactivity were physiological and unable to be impacted by any potential experimenter bias. All data analysis was automated through Matlab scripts. Any aspect of this work that included any sort of subjective measure included proper blinding of experimenter.

Reporting for specific materials, systems and methods

We require information from authors about some types of materials, experimental systems and methods used in many studies. Here, indicate whether each material, system or method listed is relevant to your study. If you are not sure if a list item applies to your research, read the appropriate section before selecting a response.

Materials & experimental systems

n/a	Involved in the study	
☐	☒	Antibodies
☒	☐	Eukaryotic cell lines
☒	☐	Palaeontology and archaeology
☐	☒	Animals and other organisms
☒	☐	Clinical data
☒	☐	Dual use research of concern
☒	☐	Plants

Methods

n/a	Involved in the study	
☒	☐	ChIP-seq
☒	☐	Flow cytometry
☒	☐	MRI-based neuroimaging

Antibodies

Antibodies used	Millipore Sigma ABE457 (1:3000), clone 2G2, ascites fluid, SAB5300463 ; Abcam ab6721 (1:2000), Goat Anti-Rabbit IgG H&L (HRP), Conjugation: HRP, Polyclonal
Validation	ABE 457 validated through western blot (https://www.sigmaaldrich.com/US/en/product/sigma/sab5300463?utm_source=google&utm_medium=cpc&utm_campaign=8906396310&utm_content=91369485804&gclid=Cj0KCQjwmMayBhDuARIsAM9HM8dHEa0MQaZOa23ay4YhYSHvQ7pOCsZcRdYeH_9QeIPp1hYyOIhPYcwaApuhEALw_wcB); abcam ab6721 validated through manufacturer (validated through western blot by manufacturer (https://www.abcam.com/products/secondary-antibodies/goat-rabbit-igg-hl-hrp-ab6721.html#lb))

Animals and other research organisms

Policy information about studies involving animals; ARRIVE guidelines recommended for reporting animal research, and Sex and Gender in Research

Laboratory animals	Male and female sprague dawley rats (Envigo) arrived at 6 weeks. Surgeries were at 8 weeks old.
Wild animals	N/A
Reporting on sex	Both sexes are represented in the current study
Field-collected samples	N/A
Ethics oversight	All procedures adhered to the University of North Carolina Chapel Hill's Institutional Animal Care and Use Committee (IACUC).

Note that full information on the approval of the study protocol must also be provided in the manuscript.

Plants

Seed stocks	N/A
Novel plant genotypes	N/A
Authentication	N/A
